# Supplementary material for: Status and Magnitude of Grey Wolf Conflict with Pastoral Communities in the Foothills of the Hindu Kush Region of Pakistan
Source: Animals (Basel). 2019 Oct 11;9(10):787. doi: 10.3390/ani9100787 (PMC6826428; doi:10.3390/ani9100787)
Supplement: Supplementary file 1 [file animals-09-00787-s001.pdf]

Supplementary

HUMAN-GREY WOLF INTERACTION SURVEY

Enumerator Name: _______________ Date: _______________________________
Respondent Name: ______________ Village Name: ________________________
Education: _____________________ Age: ________________________________
Ethnic background: ______________ Occupation___________________________
How many earning members are there in the household? _______________________________
Home much agricultural land your family own? ________________ HH Size ____________________

Predator Status:

Did you sight Grey wolf in your area in the past 1 year (Jan-Dec 2016)?

Wolf	*Response*		*Nubmer of observations*	*Status*		
Sighting/observation	*Yes*	*No*		*Common*	*Rare*	*Absent*

Population of wolf you wish to increase/maintain/reduce /eliminate from your area:

↑ / → / ↓/x

Increase	*Maintain*	*Reduce*	*Eliminate*	No response
↑	→	↓	*x*	__

Which one is most dangerous for livestock, rate 4-4(from low to high):

Not dangerous	*Dangerous*	*Slightly dangerous*	*Very dangerous*	*Extremly dangerous*
0	*1*	*2*	*3*	*4*

Livestock

How many livestock your family own?

Livestock	**Goats**	**Sheep**	**Cattle**	**Other**
Number				
Vaccinated				

Other: Donkey, Horse etc.

Mortality due to Disease in 1 year (January – December 2016):

Livestock	Goats	Sheep	Cattle	Yak	Other
Number					

Livestock sold in 1 year:

Livestock	Goats	Sheep	Cattle	Yak	Other
Number					
Total Income in Rs					

Predation Losses

Predation in 1 year:

Predator	Season/ month	Location	Prey type	Prey sex	Prey Age	Guarded (Y/N)	Circumstances

Seasons: Winter (Dec-Feb), Spring (Mar-May), Summer (Jun-Aug), Autumn (Sep-Nov)

Supplementary

HUMAN-GREY WOLF INTERACTION SURVEY

Any other Damage by wildlife: ____
